# Supplementary material for: Ephedrine and pseudoephedrine in Ephedra saxatilis on the vertical altitude gradient changed in southern Tibet Plateau, China
Source: PLoS One. 2023 Aug 25;18(8):e0290696. doi: 10.1371/journal.pone.0290696 (PMC10456159; doi:10.1371/journal.pone.0290696)
Supplement: Attachment — Submitted filename: Response to Reviewer 2.doc [file pone.0290696.s002.doc]

Dear Revewer:

We submit our manuscript entitled **“****Ephedrine and pseudoephedrine in** ***Ephedra saxatilis*** **on the vertical** **altitude gradient changed in southern Tibet Plateau, China”** for publication in ***PLoS ONE***. Thank you for receiving your valuable comments, We agree with them very much and the following modifications have been made:

1. **Introduction section**

We have made a systematic arrangement in the introduction part again, emphasizing the importance of this study. Although there are many effective chemicals in *Ephedra*, ephedrine and pseudoephedrine are the most important medicinal ingredients in *Ephedra*. At the same time, the use of metabolomics to analyze *Ephedra* metabolism and find important metabolites is emphasized, which provides convenience for ecology and plateau plant research.

2. **Method section**

We describe the method in more detail, including the preparation process of the preliminary sample and the sample injection process.

3. **Discussion section**

In the discussion section, we analyze all the results more. Combining the data and changes in the natural environment, we analyzed a number of possibilities and found that altitude has an impact on the content of ephedrine and pseudoephedrine. However, we still do not know whether some environmental factors, such as soil moisture and air temperature, will affect the content of ephedrine and pseudoephedrine. Therefore, in the next study, we will expand the sampling sites and analyze the influence of climate factors on *E. saxatilis*.

Yours sincerely，

Deji
